# Phylogenetic characterization of the fusion genes of the Newcastle disease viruses isolated in Fars province poultry farms during 2009-2011

**Published:** 2014

**Authors:** Mohammad Javad Mehrabanpour, Setareh Khoobyar, Abdollah Rahimian, Mohammad Bagher Nazari, Mohammad Reza Keshtkar

**Affiliations:** 1 *Department of Virology, Razi Vaccine and Serum Research Institute, Shiraz, Iran; *; 2 *Department of Microbiology Islamic Azad University, Jahrom Branch, Jahrom, Iran; *; 3 *Department of Microbiology Islamic Azad University, Urmia Branch, Urmia, Iran; *; 4 *Department of Virology, School of Veterinary Medicine, Shiraz University, Shiraz, Iran.*

**Keywords:** F gene, Mean death time, Newcastle disease virus, Phylogenetic analysis

## Abstract

Despite routine vaccination programs against Newcastle disease (ND), sporadic cases have occasionally occurred that remain a constant threat to commercial poultry. Ten isolates of Newcastle disease viruses (NDV) from infected broiler chicken cases were obtained from various locations in Fars province during 2009-2011 and genetically analyzed using reverse transcription polymerase chain reaction (RT- PCR) with primers specific to the viral fusion (F) protein- gene. The viruses were confirmed as NDV by hemagglutination inhibition assay and RT- PCR. The isolates based on the sequence and phylogenetic analyses of partial F gene were genotypically analyzed by RT PCR. In the present investigation, the pathogenicity of NDV strains was determined by internationally recognized test mean death time (MDT). Analysis based on F gene showed that characterized isolates possess three different types of protease cleavage site motifs and appear to show maximum identities with isolates in the region. The subsequent phylogenetic analysis was implemented using MEGA and the phylogenetic tree. The results of RT-PCR and MDT showed that 10 isolates were positive for NDV, (60% velogenic, 30% mesogenic and 10% lentogenic). The results of the phylogenetic analysis showed that 10 NDV isolates from Iran belong to the class II, genotype III viruses. This information is fundamental to improve the efficacy of controlling strategies and vaccine development for NDV.

## Introduction

Newcastle disease (ND) is an important disease of poultry as it is highly contagious and responsible for a high mortality in susceptible birds.^1^ It is caused by Newcastle disease virus (NDV), which belongs to the genus *Avulavirus* in the family of *Paramyxoviridae*.^2^ This disease can be characterized by fatal respiratory and neurological lesions that lead to great economic losses worldwide, including Iran.^3^ It is an endemic and sometimes epizootic disease in chickens.^4^ Vaccines consisting of either live or inactivated viruses have been developed and applied to minimize the impact of ND. However, the incidence of this disease remains high. Newcastle disease virus has approximately a 15kb RNA genome encoding six proteins including nucleo-protein (NP), phosphoprotein (P), matrix protein (M), fusion protein (F), hemagglutinin-neuraminidase (HN), and large protein (L), respectively.^2^^,^^5^ Among them, F protein is regarded as an important pathogenic marker of NDV.^6^ In virus classification according to the *in vivo* pathogenicity test parameters the Mean Death Time (MDT) in specific pathogen free (SPF) chicken embryos and NDV can be put in to highly pathogenic (velogenic), intermediate (mesogenic), or apathogenic (lentogenic) categories.^7^^,^^8^

Using reverse transcriptase polymerase chain reaction (RT-PCR) followed by direct sequencing and analysis of the fusion protein gene cleavage site, has been performed for NDV research and surveillance.^9^^-^^12^ The F gene was chosen for sequencing because this gene is a major determinant of virulence and NDV isolates are grouped into genotypes based on the sequences of this gene.^13^ The NDV particles contain a glycoprotein precursor, F_0_, consisting of 553 amino acids, which has to be cleaved to F_1_ and F_2_ polypeptides that are disulphide linked by proteases in host cells, allowing the virus particles to become infectious.^10^ Thus, analyzing the sequence of the F protein cleavage site can aid in pathogenicity prediction and diagnosis of NDV virulence.^14^ NDV with at least three arginine or lysine residues within the residues 113-116 at the C-terminus of F2 protein and a Phenylalanine at residue 117 at the N-terminus of the F_1_ protein are considered as a virulent strain.^15^ Phylogenetic analysis of NDV is a powerful mean for investigating epidemiological relationships among the NDV isolates present in various parts of the world. Thus, in this study 10 isolates of NDV were collected from broiler chicken farms in Fars province, Iran. In this study, the isolates were genotypically analyzed using RT-PCR with specific primers to viral F gene (535 bp) and the obtained sequences were compared to the previously published sequences.

## Materials and Methods

**Viruses. **In the present study, we determined the F gene sequences of 10 NDV strains isolated from outbreaks in broiler chicken farms in Fars province, Iran. The infected chickens showed severe neurological and/or respiratory symptoms. The viruses were confirmed as NDV by hemagglutination inhibition assay using standard NDV serum. Initial isolation of the viruses was performed on 10-day-old SPF embryonated chicken eggs (ECE).^14^ Serological identification was confirmed by RT-PCR with specific primers.^16 ^Positive samples were kept at –70 ˚C until further use.

**Biologic pathotyping. **The virulence level of each isolate was initially measured by MDT of chicken embryos and RT-PCR.^7^

**Preparation of viral RNA. **Collected viruses, from the allantoic fluid (150 µL) of SPF embryonated chicken eggs inoculated with the NDV, were used as the source of viral RNA. The RNA extraction was performed using VeTeK^TM^ viral gene spin kit (Intron Biotechnology Inc., Seongnam, Korea), according to the manufacturer’s recommended protocol. The viral RNA was resuspended in DNase/RNase free water and stored at –70 ˚C until use.

**Reverse transcription-polymerase chain reaction. **For genotypically analyzing of the virus isolates, one-step RT-PCR using pre mix kit (INtRON Biotechnology Inc., Seongnam, Korea) was performed. The PCR amplification and sequencing were performed using degenerative primers (Forward Primer: 5´-ATGGGC(C/T)CCAGA(C/T) CTTCTA-3´; and Reverse Primer: 5´-CTGCCACTGCTAGTT GTGATAATCC-3´) specific to fusion (F) protein gene.^17^ The primers generated an expected amplicon size of 535 bp (nucleotide 47-535) fragment spanning the regions between nucleotides 47 and 581 of the fusion protein, including the F_0_ cleavage site standard. RT-PCR was carried out in 8 µL reaction mixture using Vetek viral gene-spin Viral DNA/RNA Extraction Kit (Intron Biotechnology Inc., Seongnam, Korea) containing (dNTP, enzyme mix, reaction buffer and stabilizing buffer), 1 µL from each forward and reverse primer (25 µM), 2 µL of template RNA, 0.25 RNase inhibitor and 8 µL of RNase free water was added to make a reaction volume of 20 µL.^18^ The cycling parameters for PCR reaction were 48 ˚C for 45 min at reverse transcription (RT), and 35 cycles of 94 ˚C for 2 min, 56 ˚C for 2 min, and 72 ˚C for 1 min, followed by 72 ˚C for 10 min.^19^ The amplicons were separated in 1.5% agarose gel electrophoresis and visualized under ultra-violet light after being stained with ethidium bromide.^19^

**Purification and sequencing of PCR products. **The PCR products were purified by high pure PCR product purification kit (Roche, Mannheim, Germany) following the manufacturer’s instructions. Sequencing was performed by CinaGene Company in Tehran, Iran.

**Sequence alignment and phylogenetic analysis. **The nucleotide sequence of the NDV isolates, based on a portion of the F gene cleavage site, was processed by BioEdit software (Version 7.0; Isis Pharmaceuticals, Carlsbad, USA) and Clustal W multiple alignment method, the subsequent phylogenetic analysis was implemented using MEGA software (Version 4.0; Biodesign Institute, Tempe, USA). The phylogenetic tree was constructed of the representative strains from each genotype and different geographical areas were retrieved from GenBank.

## Results

**The MDT evaluation. **The MDT values of these isolates ranged from 36 to more than 90 hr, therefore, the isolates were shown that we have all three pathotypes among isolated viruses. The phylogenetic analysis placed 10 NDV isolates from Iran during 2009 to 2011 comparing the nucleotide sequences with JX131351, JQ344320, AB070396, DQ074638, EU049546, DQ439947, AJ249516, AF532752, EF565070, U22290, U22292, U22273, U13967, AF139159, AF139150, AF139133, DQ455008 and EU518685 isolates that have been published in GenBank. The RT-PCR amplified the desirable fragment of F gene of Newcastle viruses, and then amplified products were sequenced. All the viruses, evaluated by RT-PCR followed with nucleotide sequencing, contained a virulent fusion protein cleavage site represented by the motifs ^112^RRQKRF^117 ^and ^112^GRQGRL^117^.

**Phylogenetic analysis. **The results of the phylogenetic analysis showed that 10 NDV isolates from Iran in 2009 to 2011 were belonged to the class II, genotype III, (Table 1).

**Table 1:** List of 10 NDV virus isolates collected in Fars province between 2009 and 2011 and their deduced amino acid sequence at the cleavage site

**No.**	**Isolate**	**Virulence**	**F protein cleavage site**	**Genotype**
**1**	NDV/chicken/Fars/1/2009	Mesogenic	^112^GRQGRL^117^	**III**
**2**	NDV/chicken/Fars/2/2009	Velogenic	^112^RRQKRF^117^	**III**
**3**	NDV/chicken/Fars/3/2010	Velogenic	^112^RRQKRF^117^	**III**
**4**	NDV1/chicken/Fars/4/2010	Velogenic	^112^RRQKRF^117^	**III**
**5**	NDV/chicken/Fars/5/2010	Velogenic	^112^RRQKRF^117^	**III**
**6**	NDV/chicken/Fars/6/2010	Velogenic	^112^RRQKRF^117^	**III**
**7**	NDV/chicken/Fars/7/2009	Velogenic	^112^RRQKRF^117^	**III**
**8**	NDV/chicken/Fars/8/2011	Mesogenic	^112^GRQGRL^117^	**III**
**9**	NDV/chicken/Fars/9/2011	Mesogenic	^112^GRQGRL^117^	**III**
**10**	NDV/chicken/Fars/10/2011	Lentogenic	^112^GRQGRL^117^	**III**

**Fig. 1:**
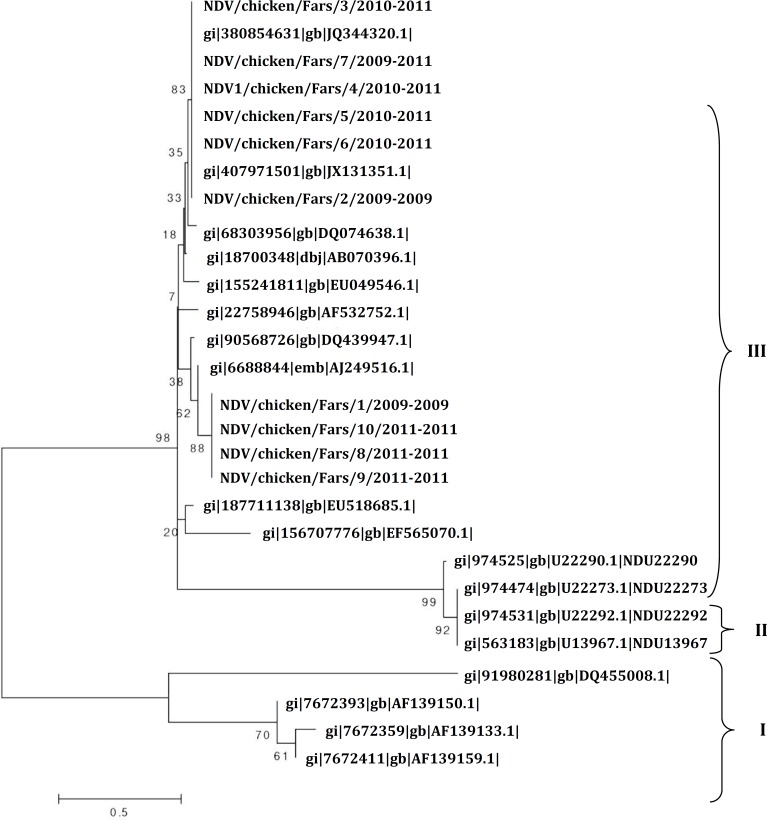
Phylogenetic relationship among 10 NDV isolates on the basis of F gene nucleotide sequences between position 47 and 435. Sequences were obtained either from the present study or GenBank. The phylogenetic tree was constructed by joining method on MEGA.

Phylogenetic relationships of the F protein of isolated Newcastle disease viruses isolated in Iran compared to other NDV sequences in GenBank. Phylogenetic tree was generated by MEGA (neighbor-joining analysis method). Numbers below branches indicate boot-strap values from 1000 replicates. Analysis was based on nucleotides 47-435, (Fig. 1). In fact, six out of 10 isolates were velogenic (< 60 hr), three out of 10 isolates were mesogenic (60 to 90 hr) and 1 of them was lentogenic (> 90 hr) according to the definition by OIE. Likewise, nine out of 10 viral isolates, evaluated by RT-PCR followed with nucleotide sequencing, contained a virulent fusion protein cleavage site represented by the motifs ^112^RRQKRF^117 ^and ^112^GRQGRL^117^. 

The results of RT-PCR and MDT showed that 10 isolates were positive for NDV, (60% velogenic, 30% mesogenic and 10% lentogenic).

## Discussion

In the present investigation the molecular characterization and phylogenetic analysis of NDVs isolated from recent outbreaks (2009 to 2011) in Fars province, Iran are shown. Newcastle disease is considered as the most important infection in the Asian countries including Iran that leads to substantial production losses. Traditionally, the pathogenicity of any new NDV isolate has been assessed by biologic methods such as MDT.^7^^,^^20^In this study, the F protein of 10 Iranian isolates revealed 98 to 100% homology for nucleotide and amino acid with class II, genotype III ND viruses. No insertion or deletions were found on these sequences. Sequence analysis of the F protein cleavage site can be used to predict potential pathogenicity of NDV which is consistent with the conventional methods such as mean death time.^10^ Low pathogenic viruses can only be cleaved by trypsin like proteases, secreted in the respiratory and intestinal tract, causing localized infection. In contrast the virulence of NDV is known to be associated with different amino acid sequence surrounding the post-translational cleavage site of the F_0_ protein, which is needed to be cleaved by the host cell proteases for induction of infection and not dependent upon trypsin for cleavage, but can be cleaved by furin which recognizes the multibasic cleavage site.^13^ Most viruses that are virulent for chickens have the amino acid sequence 112-R/KR-Q-K/R-R-116 at the C-terminus of the F_2 _protein and F (phenylalanine) at residue 117, the N-terminus of the F_1_ protein. In contrast, viruses of low virulence have sequences in the same region of 112-G/E-K/R-Q-G/E-R-116 and L (leucine) at residue 117 according to the definition of OIE.^14^ Characterization of the cleavage site is important because it is directly related to the virulence of NDV strains. Nowadays, molecular methods based on RT-PCR, nucleotide sequencing, and prediction of the amino acid sequence at the F protein cleavage site are used to determine the virulence of new isolates and for phylogenetic studies.^9^^,^^11^^,^^16^^,^^21^^,^^22^ The results of the present study showed that six of the Fars province isolates carried the ^112^PRQKRF^117^ motif and four of isolates carried ^112^GRQGRL^117^ motif which were assigned as velogenic and lentogenic motifs, respectively. Based on the phylogenetic analysis, it can be postulated that four isolates were similar lasota vaccine strain that used in chicken farms in Fars province. Phylogenetic analyses not only can be applied to pathotype prediction of NDV isolates, but also may be used to determine viral origins.^11^ The phylogenetic analysis demonstrated that NDV isolates in this study were categorized in two groups corresponding to the different genotypes, group II include genotypes I, II and III (Fig. 1). Based on our phylogenetic analysis, it could be postulated that 10 isolates were similar to JX131351, AJ249516 strains.

On the basis of nucleotide sequence analysis, isolates from Fars province seem to show maximum similarity with the isolates in the local area such as NDV/ chicken/Iran-Tehran/2010, NDV/chicken/Iran-Mashhad/ 2011 and NDV/rooster/India/2000. Our results specified that there are velogenic NDVs circulating in Fars commercial flocks and causing outbreaks in poultry industry. Phylogenetically, the similarity of NDVs of our study with other Iranian strains indicated that the new strains of NDV are present in Iran, and these isolates have being continued to circulate among commercial flocks consequently and threaten the poultry industry of Iran. Our results showed that it is possible that NDV strains isolated from Fars province could represent the strains circulating in the chicken population in other province in Iran. However, determination of the complete genome sequences of Fars province is necessary to understand the genetic relatedness among NDV strains circulating in different parts of the Iran. 

Although the close genetic relatedness of NDV strains isolated in 2009, 2010 and 2011 in another province in Iran suggest that these strains are endemic in the chicken farms in this province. On the other hand, NDV vaccine used in the broiler chicken farms may not be very effective in stopping viral shedding, which will allow unnoticed circulation of the virulent virus in the vaccinated chicken until development of an outbreak. 
